# Interleukin-10 Production by T and B Cells Is a Key Factor to Promote Systemic *Salmonella enterica* Serovar Typhimurium Infection in Mice

**DOI:** 10.3389/fimmu.2017.00889

**Published:** 2017-08-02

**Authors:** Geraldyne A. Salazar, Hernán F. Peñaloza, Catalina Pardo-Roa, Bárbara M. Schultz, Natalia Muñoz-Durango, Roberto S. Gómez, Francisco J. Salazar, Daniela P. Pizarro, Claudia A. Riedel, Pablo A. González, Manuel Alvarez-Lobos, Alexis M. Kalergis, Susan M. Bueno

**Affiliations:** ^1^Millennium Institute on Immunology and Immunotherapy, Departamento de Genética Molecular y Microbiología, Facultad de Ciencias Biológicas, Pontificia Universidad Católica de, Chile Santiago, Chile; ^2^Millennium Institute on Immunology and Immunotherapy, Departamento de Ciencias Biológicas, Facultad de Ciencias Biológicas, Universidad Andrés Bello, Santiago, Chile; ^3^Millennium Institute on Immunology and Immunotherapy, Departamento de Ciencias Biológicas, Facultad de Ciencias Biológicas y Medicina, Universidad Andrés Bello, Santiago, Chile; ^4^Departamento de Gastroenterología, Facultad de Medicina, Pontificia Universidad Católica de, Santiago, Chile; ^5^Departamento de Endocrinología, Facultad de Medicina, Pontificia Universidad Católica de Chile, Santiago, Chile

**Keywords:** *Salmonella enterica* serovar Typhimurium, interleukin-10, T cells, B cells, dendritic cells, macrophages, systemic infection, regulatory T cells

## Abstract

*Salmonella enterica* serovar Typhimurium (*S*. Typhimurium) is a Gram-negative bacterium that produces disease in numerous hosts. In mice, oral inoculation is followed by intestinal colonization and subsequent systemic dissemination, which leads to severe pathogenesis without the activation of an efficient anti-*Salmonella* immune response. This feature suggests that the infection caused by *S*. Typhimurium may promote the production of anti-inflammatory molecules by the host that prevent efficient T cell activation and bacterial clearance. In this study, we describe the contribution of immune cells producing the anti-inflammatory cytokine interleukin-10 (IL-10) to the systemic infection caused by *S*. Typhimurium in mice. We observed that the production of IL-10 was required by *S*. Typhimurium to cause a systemic disease, since mice lacking IL-10 (IL-10^−/−^) were significantly more resistant to die after an infection as compared to wild-type (WT) mice. IL-10^−/−^ mice had reduced bacterial loads in internal organs and increased levels of pro-inflammatory cytokines in serum at 5 days of infection. Importantly, WT mice showed high bacterial loads in tissues and no increase of cytokines in serum after 5 days of *S*. Typhimurium infection, except for IL-10. In WT mice, we observed a peak of *il-10* messenger RNA production in ileum, spleen, and liver after 5 days of infection. Importantly, the adoptive transfer of T or B cells from WT mice restored the susceptibility of IL-10^−/−^ mice to systemic *S*. Typhimurium infection, suggesting that the generation of regulatory cells *in vivo* is required to sustain a systemic infection by *S*. Typhimurium. These findings support the notion that IL-10 production from lymphoid cells is a key process in the infective cycle of *S*. Typhimurium in mice due to generation of a tolerogenic immune response that prevents bacterial clearance and supports systemic dissemination.

## Introduction

The infection caused by *Salmonella enterica* has a great impact in public health, both in industrialized and developing countries, because it is one of the most common causes of food-borne illness and a major cause of acute gastrointestinal ailment (1–3). Furthermore, some *Salmonella* serovars, such as *S. enterica* serovar Typhi (*S*. Typhi), are highly virulent for humans. *S*. Typhi is a host-restricted serovar that causes typhoid fever in humans due to oral ingestion of the bacteria through contaminated food or water. The disease caused by *S*. Typhi in humans is not related initially to gastrointestinal symptoms, but it is characterized by fever, bacteremia, and hepato-splenomegaly. In the absence of appropriate treatment, it can lead to intestinal hemorrhage or perforation (4, 5). Importantly, some patients become asymptomatic carriers and harbor the bacteria in gallbladder (6–8). One hallmark of *S*. Typhi infection is its ability to prevent the immune response activation to sterilize infected tissues. Thus, understanding the interaction of this bacterium with the host immune response is relevant to design more efficacious vaccines and immunotherapies to prevent this life-threatening disease.

Recently, a study performed in healthy volunteers highlighted the role of regulatory T cells (Treg cells) in the outcome of *S*. Typhi infection. This study showed that a single oral infection with this bacterium increases the amount of circulating Treg cells in peripheral blood (9). Interestingly, among the volunteers included in the study, those who developed typhoid disease symptoms expressed higher levels α4β7 integrin in Treg cells pre-challenge (9). This specific integrin is gut-homing molecule, probably accounts for the location of Treg cells in the intestine upon *S*. Typhi infection. Because one of the main molecules involved in the generation of Treg cells is interleukin-10 (IL-10), it is possible that this cytokine is involved in the ability of *S. enterica* to avoid the immune response activation. IL-10 is an anti-inflammatory cytokine, the general effects of which seems to be oriented at reducing inflammatory immune responses and prevents tissue damage (10, 11). IL-10 is secreted by cells of the innate and adaptive immune system, such as macrophages, DCs, myeloid-derived suppressor cells, neutrophils, B cells, and T cells (12). It has been suggested that by acting on antigen-presenting cells, i.e., macrophages and DCs, IL-10 can inhibit the development of Th1 type immune responses, reduce NK cell responses, prevent the differentiation of naïve T cells into effector cytotoxic T cells, and dampen the secretion of pro-inflammatory cytokines, such as IL-12 (13). Furthermore, IL-10 induces Treg cell proliferation, promoting an equilibrated immune response that control pathogen infection, altogether reducing excessive inflammatory damage to the affected tissues (14, 15).

Infection caused by *S*. Typhimurium in mice resembles the disease caused by *S*. Typhi in humans and, thus, this murine model provides a valuable tool for evaluating the infectious cycle and the immune response related to systemic *Salmonella* infection. C57BL/6 are highly susceptible to *S*. Typhimurium infection, causing 100% mortality after 10–14 days of an oral infection. Furthermore, in these animals, no specific T cell activation can be detected after infection (16–20). Therefore, *S*. Typhimurium might promote the activation of anti-inflammatory or tolerogenic mechanisms in these mice to favor systemic infection without immune response activation. Previous studies have shown that the innate IL-10 production by B cells is required by *S*. Typhimurium to cause systemic infections in mice, since transfer of B cells from mice unable to produce IL-10 increases their resistance to the infection (21). This study also suggested that B cell stimulation by *Salmonella* LPS, *via* TLR4 is a major stimulus for IL-10 production by these cells to promote systemic infection (21). Other studies have described that co-infection of *S*. Typhimurium with *Plasmodium falciparum* enhances the capacity of the bacteria to produce a systemic infection due to the increased production of IL-10 by the host, induced by this parasite (22, 23). In agreement with the role of IL-10 in *S*. Typhimurium infection, a reduced capacity of *S*. Typhimurium to cause systemic infection was described in mice treated with a neutralizing antibody that blocks the function of IL-10 (24). Interestingly, studies assessing persistent pathogens, such as *Leishmania major* (25), human cytomegalovirus (26), or *Mycobacterium tuberculosis* (27), have demonstrated that the absence of IL-10 leads to a better clearance of these pathogens, with variable degrees of immunopathology.

In this study, we demonstrate that the active induction of IL-10 production by both, B and T cells during *S*. Typhimurium infection is fundamental to generate a systemic infection in C57BL/6 mice. Our results support the notion that *S*. Typhimurium infection promotes the generation of a tolerogenic immune response characterized by an increased production of IL-10 in the intestine, spleen, and liver, preventing clearance of the bacteria from infected tissues.

## Materials and Methods

### Ethics Statement

All the experiments using mice were conducted in agreement with the ethical standards and according to the local animal protection law. All experimental protocols were reviewed and approved by the Scientific Ethical Committee for Animal and Environment Care of the Pontificia Universidad Católica de Chile (Protocol number CBB-208/2013).

### Bacterial Strains and Culture Conditions

*S*. Typhimurium ATCC14028 strain was originally obtained from American Type Culture Collection and kindly provided by Dr. Carlos Santiviago (Universidad de Chile, Santiago, Chile). *S*. Typhimurium aliquots were stored at −80°C in Luria-Bertani (LB) broth (tryptone 1%, yeast extract 0.5%, and NaCl 0.5%) supplemented with 20% glycerol. To perform the infection assays, aliquots were thawed and then grown with agitation at 37°C in LB broth until OD_600_ equal to 0.6. Then, bacterial doses were resuspended in sterile phosphate-buffered saline (PBS). All experimental protocols that included biohazards were reviewed and approved by the Scientific Ethical Committee for Research Safety of the Pontificia Universidad Católica de Chile (Protocol number CBB-208/2013).

### Mice Strains and Infection Protocol

Six- to eight-week-old C57BL/6 wild-type mice (WT), C57BL/6 IL-10^−/−^ mice (IL-10^−/−^), C57BL/6 IL-10/GFP reporter mice (Vert-X), and C57BL/6 Rag.1^−/−^ mice were originally purchased from Jackson Laboratories (Bar Harbor, ME, USA) and maintained in the pathogen-free animal facility at the Facultad de Ciencias Biológicas, Pontificia Universidad Católica de Chile. For intragastric infection (i.g), mice were anesthetized with a Ketamine 16%/Xylazine 4% solution and 1 × 10^5^ CFU of *S*. Typhimurium were administered by i.g gavage in 200 µl of PBS. For intraperitoneal infection (i.p), 1 × 10^3^ CFU of *S*. Typhimurium were injected in 100 µl of PBS in the peritoneal cavity. Survival, weight changes, and clinical symptoms were evaluated daily and classified according their clinical score (described in Table S1 in Supplementary Material).

### Bacterial Load Quantification

To evaluate bacterial loads in organs, mice were euthanized 5 days post infection and spleen, liver, and ileum were recovered and homogenized. The homogenized samples were serially diluted in PBS and plated on LB or McConkey agar plates. All colony forming units (CFUs) were quantified 24 h later and normalized based on organ weight.

### Cell Preparation and Flow Cytometry

To evaluate the IL-10 production by different cells during *S*. Typhimurium infection, IL-10/GFP VertX mice were infected and euthanized at 5 days post infection. Spleens, mesenteric lymph nodes, livers, and small intestine were collected and single-cell suspensions were prepared by homogenization through a 70 µm cell strainer (BD Biosciences). Liver samples were cut in small pieces and treated with Krebs Ringer solution plus collagenase IV (20 UI Heparin, 20.1 mM HEPES, 2.5 mM de CaCl_2_, 2 mM de MgCl_2_, collagenase IV a 250 UI/ml) during 30 min at 37°C on a rotary shaker. Then, liver tissue suspension was incubated with buffer PEBD (PBS 1× pH7.2, 2 mM EDTA, 0.5% BSA, and 1,500 U/ml DNase I) and, subsequently, processed for flow cytometry as previously described (28). For small intestine cells isolation, tissue was carefully removed of fecal and fat contents and detached of their mesentery and Peyer’s patches. Small intestine was opened longitudinally, washed thoroughly with 2% FCS HBSS, and cut into 0.5 cm segments. Sections were incubated twice in HBSS with 2 mM EDTA for 20 min at 37°C on a rotary shaker. After each incubation step, supernatant was removed, along with epithelial cells and debris. The remaining tissue was incubated for 45 min in RPMI 1640 medium supplemented with 10% fetal calf serum (FBS), 2 mM glutamine, 1 mM non-essential aminoacids, 1 mM pyruvate, 1 mM HEPES, and 1 mg/ml collagenase VIII (Sigma-Aldrich) at 37°C in agitation. Cell suspensions were finally passed through a 40-µm cell strainer and pelleted for labeling flow cytometry. Myeloid and lymphoid cells were characterized using specific surface markers: anti-Ly6C-BV605 (clone AL-21; BD PharMingen), anti-MHC-II-BV650 (clone M5/114.15.2; BD PharMingen), anti-CD24-BV421 (clone M1/69; BD PharMingen), anti-CD45-BV786 (clone 30-F11; BD PharMingen), anti-CD11c-PE-Cy7 (clone N418; BD PharMingen), anti-CD103-PerCP-Cy5-5 (clone M290, BD PharMingen), anti-CD11b-PE (clone M1/70; BD PharMingen), anti-Ly6G-AF700 (clone 1A8; BD PharMingen), anti-CD64-AF647 (clone X54-5/7.1; Biolegend), anti-CD8-BV605 (clone 53-6.7; BD PharMingen), anti-CD4-BV650 (clone RM4-5; BD PharMingen), anti-CD3-PerCP-Cy5.5 (clone 145-2C11; BD PharMingen), anti-CD19-PE (clone 6D5; BD PharMingen), anti-CD1d-BUV395 (clone 1B1; BD PharMingen). All FACS samples were first stained with LIVE/DEAD fixable Viability Stain 510 (BD PharMingen). The data were obtained in LSR Fortessa X-20 Flow cytometry (BD Biosciences) and analyzed in Flow Jo software V7.0. The gating strategy to identify myeloid and lymphoid GFP^+^ cells is shown in Figures S1 and S2 in Supplementary Material, respectively.

### Intracellular Staining for Detection of IL-10 and FoxP3 by Flow Cytometry Analyses

Six- to eight-week-old female C57BL/6 WT mice were anesthetized and challenged i.g. with either 1 × 10^5^ CFU of *S*. Typhimurium or an equal volume of PBS (as uninfected control). At day 4 post-infection, mice were euthanized and spleens, livers, and small intestine were collected and single-cell suspensions were prepared for flow cytometry, as described above. Cells suspensions were stimulated with 10 µg/ml brefeldin A (Sigma-Aldrich) for 5 h prior to intracellular staining. Then, cells were stained with anti-CD45-BV786 (clone 30-F11; BD PharMingen), anti-TCR-β-PE-Cy7 (clone H57-597; BD PharMingen), anti-CD4-APC-H7 (clone RM4-5; BD PharMingen), anti-CD25-BV421 (clone PC61; BD PharMingen). After staining, cells were fixed and permeabilized (Cytofix/Cytoperm kit; BD PharMingen) and, subsequently, stained intracellularly with anti-IL10 (clone JES5-16E3, BD PharMingen) and anti-Foxp3 (clone PE-CF594, BD PharMingen). All FACS samples were first stained with LIVE/DEAD fixable Viability Stain 510 (BD PharMingen). The intracellular stain was controlled by FMO and the analysis was performed using a LSR Fortessa X-20 Flow cytometry (BD Biosciences).

### Multiplex Cytokine Assay

Blood was collected by cardiac puncture from IL-10^−/−^ and WT mice infected i.g with 1 × 10^5^ CFU of *S*. Typhimurium. Total blood collected was mixed with 100 µl of heparin (125 UI/ml), maintained during 15 min at 37°C and centrifuged at 3,000 × *g* for 15 min. Serum was collected and stored at −80°C until used. Levels of IL-1β, IL-6, IL-10, IFN-γ, IL-12p70, IL-23p19, and TNF-α were measured on a Luminex 200 (Merck Millipore), using a mouse magnetic luminex screening assay (R&D systems), according to manufacturer instructions.

### Quantitative Real-time PCR

RNA from different organs of mice was purified using the SV Total RNA Isolation System (Promega), according to the manufacturer’s instructions. The same amount of messenger RNA (mRNA) for each sample was used to made the reverse transcription and PCRs, using TaqMan RNA-to-Ct 1-step kit (Applied Biosystems). Murine interleukin 10 (*il-10*; Assay ID: mm00439614_m1), transforming growth factor-β (*tgf-*β; Assay ID: mm01178820_m1), forkhead box P3 (*foxp3*; Assay ID: mm00475162_m1), cytotoxic T-lymphocyte antigen 4 (*ctla-4*; Assay ID: mm01253995_m1), programmed cell death protein 1 (*pd-1*; Assay ID: mm01285676), and β-2-microglobulin (β*2m*: Assay ID: mm00437762) taqman probes (Applied Biosystems) were used to amplify RNA. Quantitative real-time RT-PCRs (qRT-PCRs) were carried out using a StepOne plus thermocycler (Applied Biosystems), with the following cycling conditions: 1 cycle of 48°C for 15 min and 95°C for 10 min, followed by 40 cycles of 95°C for 15 s and 60°C for 1 min. For all tested samples, the expression of the target and the housekeeping gene (β*2m*) was calculated. The mRNA gene expression of every sample was normalized by dividing the mRNA value of the target gene with the appropriated β*2m* mRNA value. After the standardization, the abundance of each target mRNA was determined by the comparative method (2^−ΔΔct^) of StepOne software. All samples were analyzed at least by triplicate.

### T and B Cell Isolation and Stimulation

Mice were euthanized as described above and spleen from either IL-10^−/−^ or WT mice were recovered. Total spleen cells were centrifuged at 300 × *g* for 10 min at 4°C and red blood cells were lysed using ACK buffer. Cells were washed, resuspended in RPMI 1640 medium supplemented with 10% FBS, and counted by trypan blue staining. B and T cells were purified by negative selection using the Pan B and T cells isolation kit, Miltenyi Biotec, respectively, according to manufacturer instructions. Isolated cells were counted by trypan blue staining and resuspended in RPMI medium to a concentration of 1 × 10^6^ cells/ml. For cytokine production, 1 × 10^6^ B or T cells were placed in eppendorf tubes and infected with *S*. Typhimurium (MOI:25) in antibiotic-free RPMI-1640 medium for 1 h at 37°C. Cells were washed and treated with gentamicin (100 µg/ml) for 1 additional hour at 37°. Culture supernatants were collected after 24 h and stored at −80°C until used. In addition, purified B cells were stimulated either with LPS (1 µg/ml, *S*. Typhimurium; Sigma, St. Louis, MO, USA), or with anti-IgM antibody (10 µg/ml) for 24 h, or both. Similarly, purified T cells were stimulated over plates coated with anti-CD3/CD28 antibody (1 µg/ml) for 24 h. Levels of IL-10 were measured in the supernatants of these cells using an ELISA Kit II (BD Biosciences).

### Cell Adoptive Transfer Experiments

For adoptive transfer studies, T and B cells were purified from the spleen of C57BL/6 WT mice using a magnetic cell-sorting column (MACS) according to the manufacturer’s instructions (Pan T and B cells isolation kit, Miltenyi Biotec). DCs and bone marrow-derived macrophages were differentiated and cultivated from bone marrow precursors of WT mice, as described before, and MACS enriched, respectively (Miltenyi Biotec). The purified cells were adoptively transferred (1 × 10^6^ cell/mouse) by intravenous injection into IL-10^−/−^ mice or Rag.1^−/−^ recipient mice (only T and B cells). After 24 h, mice recipients were challenged *via* i.g. with 1 × 10^5^ CFU of *S*. Typhimurium as described above. Animal survival and clinical parameters of infection were recorded daily. Bacterial load was evaluated in spleen and liver at the end of each experiment.

### Statistical Analysis

Statistical analyses were performed using Prism v6 (GraphPad Software, San Diego, CA, USA). Unpaired Student’s *t*-test and Mann–Whitney *U*-test were used to assess whether the means of two normally distributed groups differed significantly. One-way ANOVA with Bonferroni’s multiple comparison post-test was used to compare multiple means. Two-way ANOVA analysis with repeated measures was also used in some experiments, with Sidak or Tukey post-test. Survival curves were compared using a Kaplan–Meier plot and log-rank test.

## Results

### Systemic *Salmonella* Infection Requires IL-10 Production in Mice

**Figure 1:**
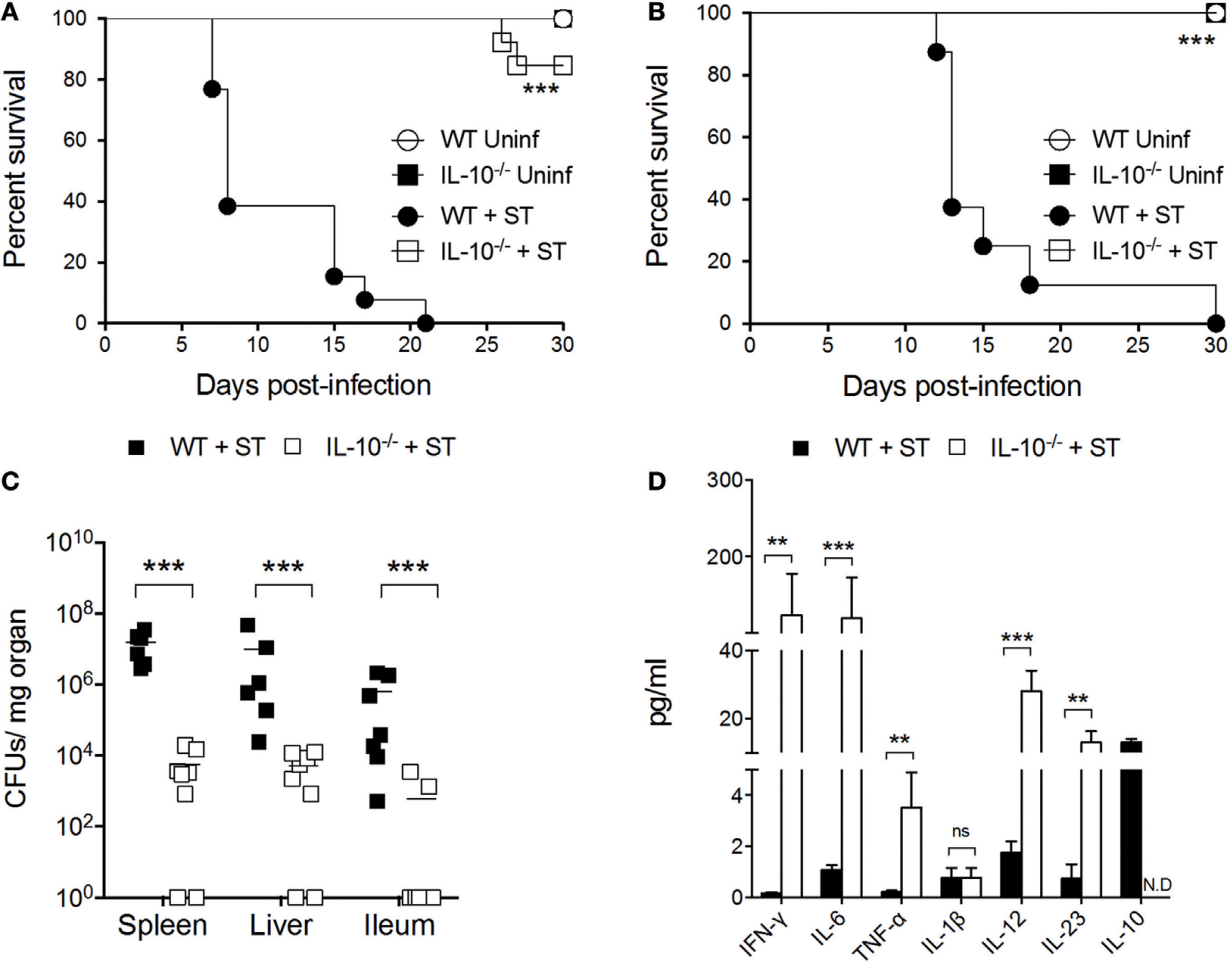
Absence of interleukin-10 (IL-10) production improves host survival and prevents systemic dissemination of *S*. Typhimurium. Wild-type (WT) C57BL/6 and IL-10^−/−^ mice were infected **(A)** orally and **(B)** intraperitoneally with *S*. Typhimurium (ST) and survival rate were followed for 30 days. **(C)** Bacterial burden in spleen, liver, and ileum and **(D)** cytokine production in serum were evaluated in orally infected WT and IL-10^−/−^ mice at 5 days post infection. For survival assays, ***P < 0.001 log-rank test. For colony forming units (CFUs) loads and cytokine quantification, a student’s *t*-test was performed between WT- and ST-infected mice; **P < 0.01; ***P < 0.001.

To evaluate how IL-10 production *in vivo* influences the outcome of *S*. Typhimurium infection, intragastric and intraperitoneal infections were performed in C57BL/6 WT and IL-10^−/−^ mice, and survival rates were evaluated daily for 30 days. As expected, we observed that in both infection models, IL-10^−/−^ mice displayed significantly higher survival rates than WT mice (Figures 1A,B). Next, to demonstrate that IL-10^−/−^ mice clear *S*. Typhimurium more efficiently than WT mice, bacterial loads were measured at 5 days post infection in ileum, liver, and the spleen. As shown in Figure 1C, IL-10^−/−^ mice showed significantly less bacterial loads in the above-mentioned organs, as compared to WT mice. The reduced bacterial colonization and better survival of IL-10^−/−^ mice correlates with higher levels of IFN-γ, IL-6, TNF-α, IL-12, and IL-23 in serum (Figure 1D). Notably, at the same time post infection, we observed that WT mice did not show an increase in these pro-inflammatory cytokines in serum, despite the high number of bacteria in the tissues. Moreover, the only cytokine that was significantly increased in the serum of the infected WT mice was IL-10. These results support the notion that IL-10 production during *S*. Typhimurium infection favors systemic infection caused by this bacterium in mice.

### IL-10-Producing Cells Reside in Ileum, Liver, and Spleen after 5 Days of *S*. Typhimurium Infection

**Figure 2:**
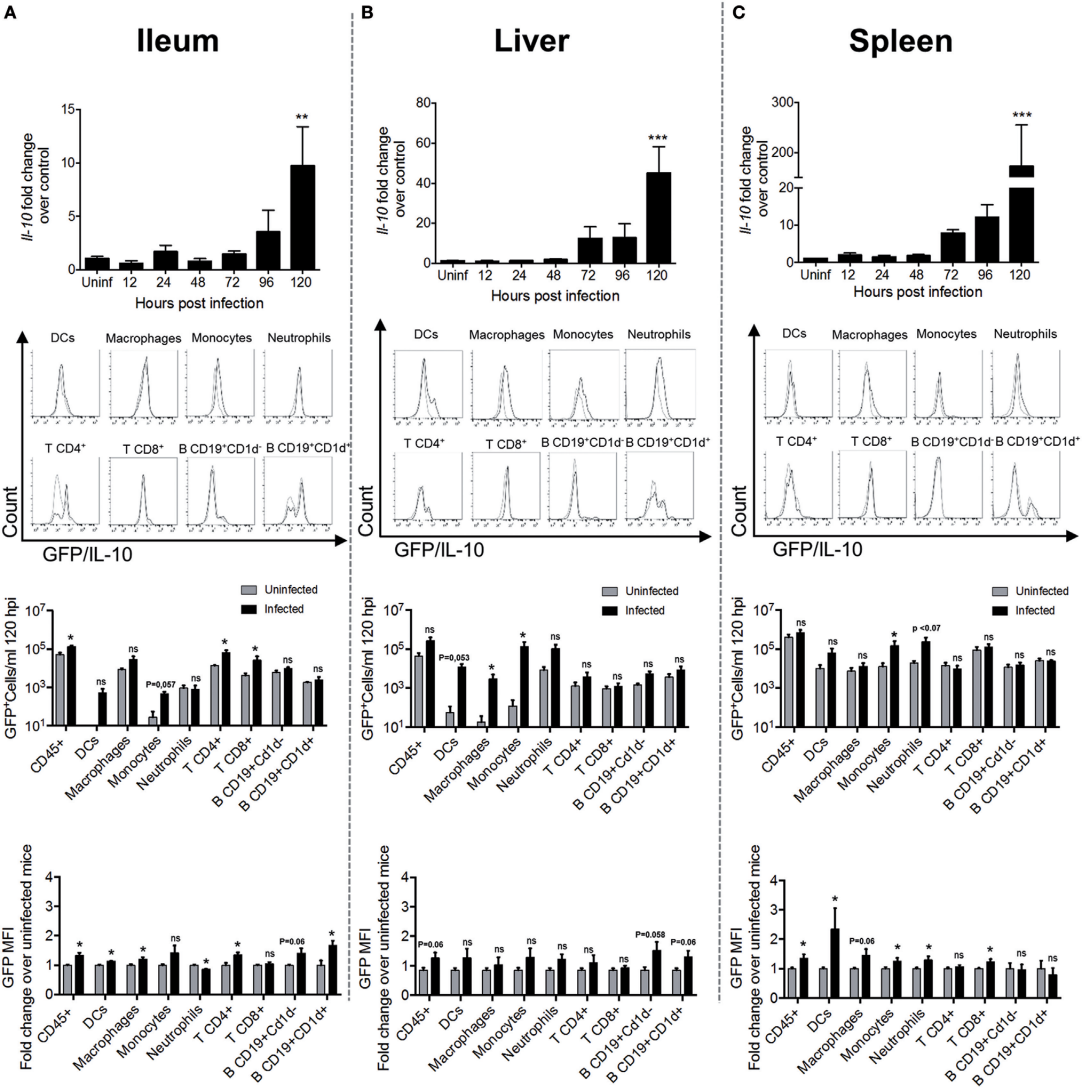
Interleukin-10 (IL-10) production significantly increases in target tissues after 120 h of *S*. Typhimurium infection. In the panels of the upper row, *Il-10* messenger RNA (mRNA) production was quantified by RT-PCR in ileum **(A)**, liver **(B)**, and spleen **(C)** of infected WT mice at different time post-infection. The panels of the second row show representative flow cytometry histograms obtained to identify the cell source of IL-10 production at 120 h post infection in ileum **(A)**, liver **(B)**, and spleen **(C)** of IL-10/GFP VertX that were orally infected by *S*. Typhimurium. In the third-row panels, a quantification of IL-10 producing total leukocytes, dendritic cells (DCs), macrophage, monocytes, neutrophils, CD4^+^ T cells, CD8^+^ T cells, CD19^+^CD1d^−^ B cells, and CD19^+^CD1d^+^ B cells in ileum **(A)**, liver **(B)**, and spleen **(C)** were identified by flow cytometry. The quantification of IL-10/GFP^+^ cells was performed using count bright beads. The last row panels show the fold change of the medium fluorescence intensity of GFP/IL-10 in myeloid and lymphoid cell types during *S*. Typhimurium infection at 120 h post infection. For *Il-10* mRNA production, **P < 0.01; ***P < 0.001 (one-way analysis of variance test with *a posteriori* multiple comparison test).

To identify when and where IL-10 is produced during *S*. Typhimurium infection in WT mice, evaluation of the mRNA encoding IL-10 was performed in the most important target tissues of this bacterium, at 12, 24, 48, 72, 96, and 120 h post oral infection with 1 × 10^5^ CFUs. As shown in Figure 2 (upper panels), IL-10 encoding mRNA was significantly increased after 120 h post infection in ileum (Figure 2A) <liver (Figure 2B) <spleen (Figure 2C). Other organs that are also *S*. Typhimurium targets, such as colon and mesenteric lymph nodes, did not show increased production of IL-10 encoding mRNA at any time evaluated (data not shown). To identify which CD45^+^ cells in ileum, liver, and spleen were the main IL-10 producers, transgenic IL-10/GFP VertX mice were infected with 1 × 10^5^ CFUs of *S*. Typhimurium and IL-10-producing cells were evaluated at 120 h post infection by flow cytometry. As shown in Figure 2, second and third panels, we observed a significantly increased number of CD45^+^IL-10-producing cells (GFP^+^ cells) in the ileum of the infected mice, as compared to uninfected mice. Importantly, our results show that the IL-10-producing cells in this tissue were mainly T cells. In the liver and spleen, although we did not detect a significantly increase of total CD45^+^IL-10-producing cells, we observed increased numbers of IL-10 producing monocytes, macrophages, and dendritic cells (DCs). Beyond the absolute cell number of IL-10-producing cells, we also quantified the intensity of IL-10 GFP^+^ expression in these CD45^+^ leukocytes after *S*. Typhimurium infection. As shown in Figure 2 (fourth panel), increased GFP^+^ mean fluorescence intensity (MFI) was observed in ileum (DCs, macrophages, and CD4^+^ T cells) and spleen (DCs, monocytes, neutrophils, and CD8^+^ T cells). These results suggest that *S*. Typhimurium infection promotes both increased number of IL-10-producing cells and increased IL-10 production by resident cells in target tissues.

**Figure 3:**
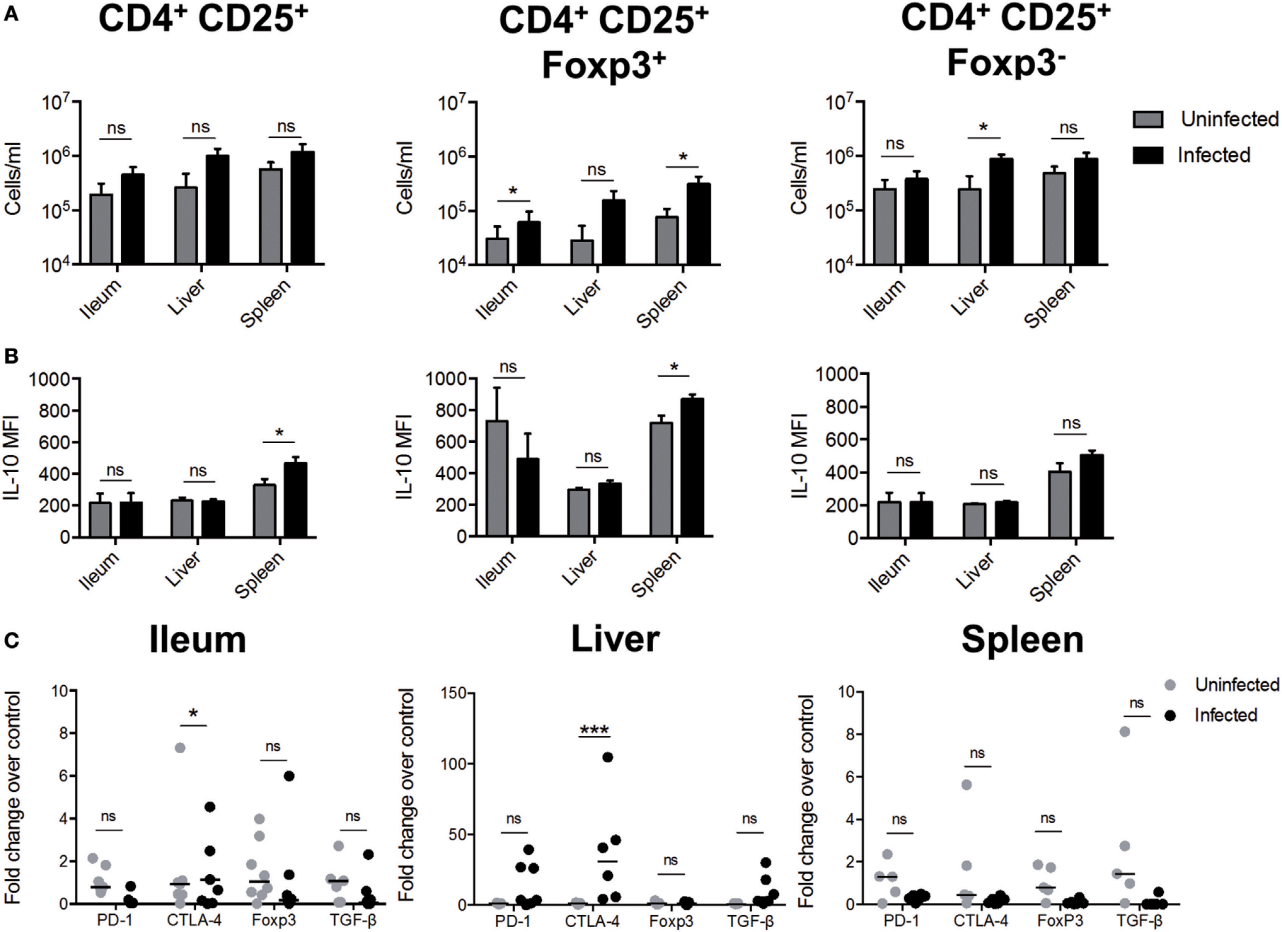
*S*. Typhimurium infection induces the generation of regulatory T cells (Tregs cells). C57BL/6 mice were left uninfected or orally infected with 1 × 10^5^ CFU of *S*. Typhimurium and after 96 h post infection of infection the ileum, liver, and spleen was recovered to detect the presence of Tregs. **(A)** Quantification of total CD4^+^CD25^+^; CD4^+^CD25^+^FoxP3^+^, and CD4^+^CD25^+^FoxP3^−^ T cells. **(B)** Measurement of mean fluorescence intensity (MFI) of IL-10 secretion by flow cytometry in target tissues ex vivo. Gray bars = uninfected; black bars = infected. *P < 0.05, ***P < 0.001 (Student’s *t*-test). **(C)** Tregs cells markers expression in target tissues during *S*. Typhimurium infection. The quantification of messenger RNA (mRNA)-encoding Tregs cells markers were evaluated in target tissues infected by *S*. Typhimurium, using quantitative real-time RT-PCR at 5 days post infection with specific TAQMAN probes. β2-microglobulin gene was used as endogenous control. The results are shown as fold change of relative expression, compared with wild-type (WT) uninfected mice (ΔΔCT). Statistical analysis was performed using two-way ANOVA with Bonferroni post test (*P < 0.05; **P < 0.01; ***P < 0.0001).

To evaluate whether *S*. Typhimurium infection induces the differentiation of Treg cells in these tissues, C57BL6 mice we orally infected with 1 × 10^5^ CFUs and 96 h post-infection were measured the amount of CD4^+^CD25^+^FoxP3^+^ and CD4^+^CD25^+^FoxP3^−^ T cells *in situ* (as described in Figure S3 in Supplementary Material). As shown in Figure 3A (left panel), no difference in the amount of CD4^+^CD25^+^ T cells was observed in *S*. Typhimurium-infected mice, as compared to uninfected mice. However, in ileum and spleen of infected mice, we observed a significantly increase in the amount of CD4^+^CD25^+^FoxP3^+^ T cells. On the other hand, in the liver, we observed a significantly increase in the number of CD4^+^CD25^+^FoxP3^−^ T cells. In addition, we observed that CD4^+^CD25^+^FoxP3^+^ T cells from the spleen also displayed an increase in MFI IL-10, suggesting that CD4^+^CD25^+^FoxP3^+^ T cells are an important source of IL-10 in the spleen (Figure 3B). We also determined the level of mRNA encoding proteins expressed in Tregs in ileum, spleen, and liver. As shown in Figure 3C, we observed increased expression of *ctla-4* in ileum and liver but not in the spleen. Although the mean value was not statistically significant, we observed increased production of *pd-1* in the liver of some infected mice. We did not observe changes in the expression of *foxP3* or *tgf-*β in total mRNA from infected tissues. In summary, our results allow us to conclude that *S*. Typhimurium induced the expression of IL-10 in specific infected organs and that the main cellular sources of this cytokine are DCs, macrophages, monocytes, and T cells located in the ileum, liver and spleen. Our results also suggest that *S*. Typhimurium infection promotes CD4^+^CD25^+^FoxP3^+^ Treg cell response in ileum and spleen, which probably favors systemic disease after *S*. Typhimurium infection.

### IL-10 Production by T Cells Restores the Susceptibility to *S*. Typhimurium Systemic Infection in IL-10^−/−^ Mice

**Figure 4:**
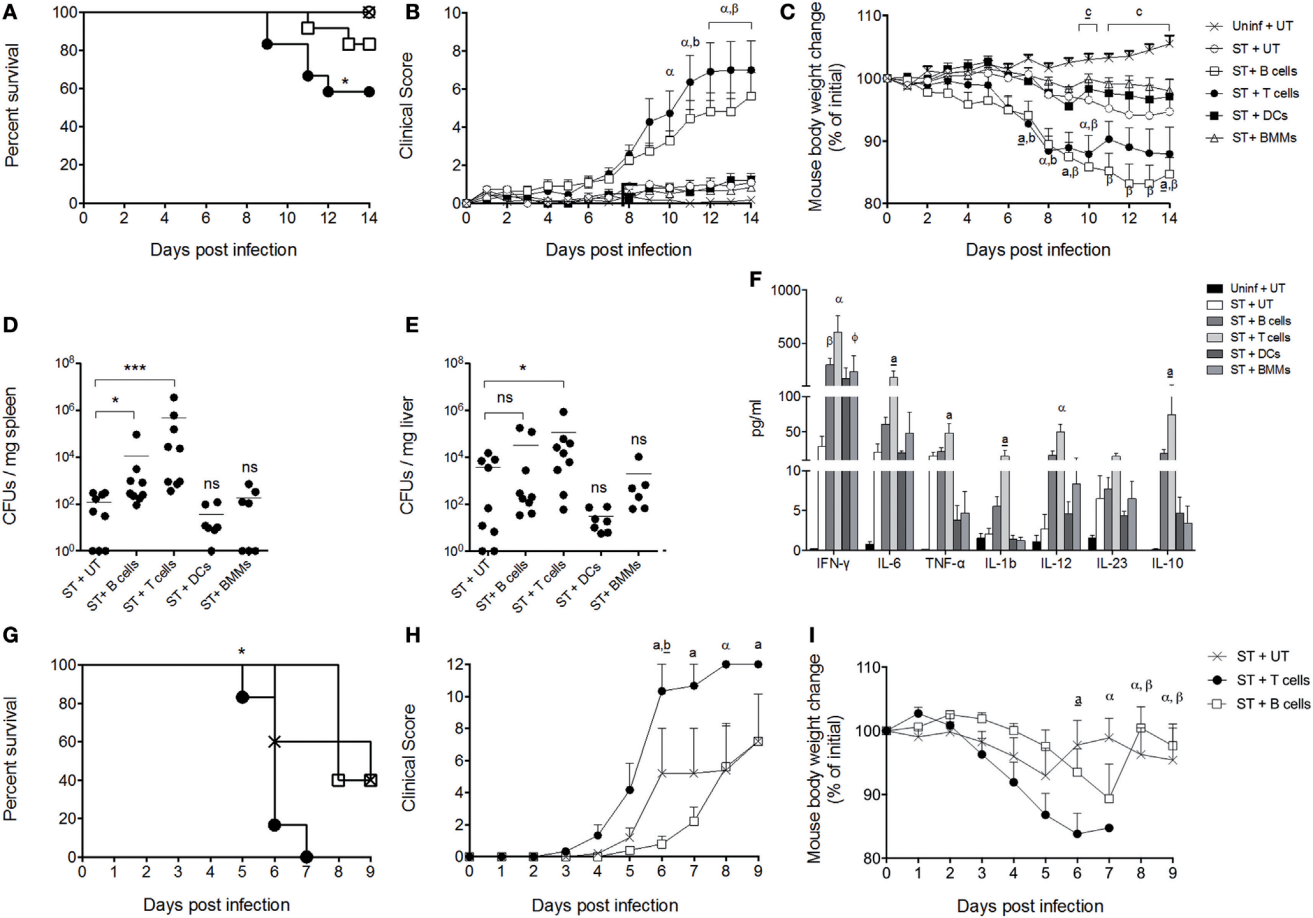
Interleukin-10 (IL-10) production by T cells enhances the severity of the infection caused by *S*. Typhimurium in mice. Dendritic cells (DCs), BM macrophages, B cells, and T cells isolated from wild-type (WT) mice (1 × 10^6^ cells) were adoptive transferred to IL-10^−/−^ recipient mice. Twenty-four hours after cell transfer, recipient mice were orally infected with 1 × 10^5^ CFU of *S*. Typhimurium. Then survival rate **(A)**, clinical score **(B)**, and weight loss **(C)** of DCs (ST + DCs), BMM (ST + BMMs), B cells (ST + B cells), and T cells (ST + T cells) recipient mice were followed over 14 days. As controls, uninfected/untransferred (Uninf + UT) and infected/untransferred (ST + UT) groups were added. After 5 days of infection, bacterial burden was evaluated in **(D)** spleen and **(E)** liver in eight randomly selected DCs, BMM, B cells, and T cell recipient mice. After 14 days of infection, serum of DCs, bone marrow-derived macrophages (BMM), B cells, and T cell recipient mice was obtained and cytokine production was evaluated by Luminex **(F)**. B cells and T cells isolated from WT mice were adoptively transferred to Rag.1^−/−^ mice. Twenty-four hours later, mice were orally infected with 1 × 10^5^ CFU of *S*. Typhimurium, and survival **(G)**, clinical score **(H)**, and weight loss **(I)** were evaluated for 9 days. For CFUs and cytokine quantification *in vivo* *P < 0.05, ***P < 0.0001 by Kruskal–Wallis test compared with ST + UT mice (IL-10^−/−^ mice infected untransferred). Statistical analysis of cytokine production is indicated by letters in the figure and was performed using two-way ANOVA with Bonferroni post-test. a: *P < 0.05; a: **P < 0.01; α,β: ***P < 0.0001; represents IL-10^−/−^ mice infected and transferred with T (a, a, α) and B cells (b, β), respectively, compared with ST + UT mice. ɸ: *P < 0.05; IL-10^−/−^ mice infected and transferred with BMMs compared with ST + UT mice. *P < 0.05 log-rank test was performed for survival curves; body weight changes and clinical score were analyzed using two-way ANOVA with Bonferroni post-test. IL-10^−/−^ mice infected and transferred with T (a, a, α) and B cells (b, b, β) were compared with ST + UT mice, respectively. Statistical significance was indicated by letters. a,b: *P < 0.05; a,b: **P < 0.01; α,β: ***P < 0.0001. Comparison between Uninf + UT mice and ST + UT mice was represented as c: *P < 0.05; c: ***P < 0.0001.

To demonstrate that the production of IL-10 by certain subsets of immune cells is key to *S*. Typhimurium to cause systemic infection in mice, IL-10^−/−^ mice were adoptively transferred with either T cells, B cells, DCs, or macrophages from WT mice that probably produce IL-10 *in vivo* after *S*. Typhimurium infection. The selection of these cells was based on previous studies showing the relevance of IL-10 production by these cells in different experimental settings of *S*. Typhimurium infection (21, 22, 24, 29–36), and according to the results showed in Figure 2, which suggest that these cells are contributing to IL-10 production in ileum, liver, and spleen at 120 h post infection. As controls, a group of uninfected mice and a group of infected mice that did not receive cells from WT mice were included. After 24 h of adoptive transfer, recipient mice were orally infected with 1 × 10^5^ CFUs of *S*. Typhimurium and survival, clinical score, and weight loss were evaluated for 14 days. As shown in Figure 4A, IL-10^−/−^ mice that received T cells showed increased mortality as compared to IL-10^−/−^ mice that received B cell, DCs, and macrophages. Accordingly, IL-10^−/−^ mice that received T cells showed a significant increase in clinical score and weight loss starting day 7 post infection, as compared to uninfected mice and mice that received IL-10-producing DCs and macrophages (Figures 4B,C). Importantly, IL-10^−/−^ mice that received B cells also showed a significant increase in clinical score and weight loss; however, the survival rate at day 14 post infection was better than IL-10^−/−^ mice that received T cells. Supporting these observations, IL-10^−/−^ mice that received T cells showed the highest bacterial loads in spleen and liver, as compared to the other infected experimental groups. IL-10^−/−^ mice recipient for B cells also showed increased bacterial loads only in spleen, as compared to infected mice that did not receive cells from WT mice (Figure 4D). To evaluate the level of IL-10 production and the amount of other pro-inflammatory cytokines in the recipient IL-10^−/−^ mice, serum was obtained from all experimental groups after 14 days of infection. As observed in Figure 4F, IL-10^−/−^ mice that received T cells showed the highest level of IL-10 in serum, followed by B-cell recipient IL-10^−/−^ mice. Although IL-10^−/−^ mice that received DCs and macrophages showed a slight increase in IL-10 in serum, the value detected was 100-time less than the levels of IL-10 found in T cells and B cell recipient IL-10^−/−^ mice. Importantly, IL-10^−/−^ mice that received T cells showed the highest level of pro-inflammatory cytokines in serum at 14 days post-infection, as compared with infected IL-10^−/−^ mice that did not receive IL-10-producing cells, which agrees with the high clinical score, weight loss, and mortality observed in these mice after infection. The above results are also in agreement with septicemia disease development in these animals.

Finally, to identify whether other IL-10-producing cells in the absence of T cells and B cells also influence the severity of *S*. Typhimurium infection, Rag.1^−/−^ mice were orally infected with 1 × 10^5^ CFUs of *S*. Typhimurium, and survival, clinical score, and weight loss were evaluated during 9 days. As shown in Figures 4G–I, these mice (ST + UT) showed a mortality rate equivalent to WT C57BL/6 mice, suggesting that IL-10 produced by other cells than T and B cells indeed influence the outcome of *S*. Typhimurium infection. However, when these mice were adoptively transferred with T cells purified from WT mice, recipient mice showed an increased mortality rate (Figure 4G), clinical score (Figure 4H), and weight loss (Figure 4I), as compared to untransferred mice. Importantly, when Rag.1^−/−^ were adoptively transferred with B cells purified from WT mice, we did not observe an increased mortality, clinical score, or weight loss, as observed for IL-10^−/−^ mice (Figures 4A–C). These results suggest that IL-10 produced by B cells is likely not enough to fully recapitulate *S*. Typhimurium infection in the absence of T cells.

To control that IL-10-producing cells secrete this cytokine after direct contact with *S*. Typhimurium infection, we performed an *in vitro* assay in which DCs, B cells, and T cells were infected with *S*. Typhimurium for 2 h and, after 24 h post treatment, the production of IL-10 was tested by ELISA. As shown in Figure S4 in Supplementary Material, the lowest level of IL-10 production after infection was observed in T cells. These results suggest that *in vivo* the direct infection of T cells by *S*. Typhimurium may not account for IL-10 production and, probably, the interaction of T cells with other *S*. Typhimurium-infected cells is required to induce the production of IL-10 by these cells in the infected host.

## Discussion

In this study, we have demonstrated the role of IL-10 production in the outcome of *S*. Typhimurium infection in mice, which supports the notion that the production of this cytokine by the host during infection is a key factor in the ability of the bacterium to cause systemic infection in susceptible hosts.

In bacterial infections, IL-10 is readily produced by cells from the innate immune system upon recognition of several pathogen-associated molecular patterns (PAMPs) (37–41). However, there are multiple pro-inflammatory cytokines also produced by immune cells during an infection, which promote an acute inflammatory response to restrict bacterial dissemination to other tissues (13, 42, 43). In the case of *S*. Typhimurium, it has been described that bacterial recognition by cells from the innate immunity, such as neutrophils, macrophages, and DCs, promotes the secretion of pro-inflammatory cytokines (44–50). However, as shown in this study, at the beginning of the systemic phase of infection, pro-inflammatory cytokines cannot be detected in the serum of WT mice infected with *S*. Typhimurium, which are highly susceptible to infection caused by this bacterium. Importantly, we observed that among the cytokines tested in this study at 5 days of infection, the only one that was elevated in the serum of infected mice was IL-10. On the other hand, the high levels of pro-inflammatory cytokines detected in the serum of infected IL-10^−/−^ mice at similar time points post infection indicate that the absence of IL-10 allows the recognition of bacteria to generate an inflammatory immune response. Importantly, we observed a high level of serum IFN-γ in infected IL-10^−/−^ mice at 5 days of infection, which is a cytokine important for the elimination of intracellular *S*. Typhimurium (51–54). These results suggest that in the absence of IL-10, PAMPs from *S*. Typhimurium can be readily recognized by the host and promote an inflammatory immune response, but in the presence of IL-10, this response seems to be totally turned off, despite the high bacterial loads found in the tissues of the infected mice.

In susceptible mice, it has been described that *S*. Typhimurium dampens the bactericidal capacity of phagocytic cells and reside intracellularly within a vacuole, aided by virulence proteins secreted to the host cells cytoplasm through the type three secretion system encoded in *Salmonella* Pathogenicity Island 2 (SPI-2). This way, the phagolysosome activity of phagocytic cells is prevented and the intracellular bacteria cannot be cleared (16, 55–58). Furthermore, antigen presentation is impaired due to lack of antigen degradation, which in turn prevents the activation of the anti-*Salmonella* adaptive immune response (19, 59–61). Likely, the production of IL-10 enhances the ability of *S*. Typhimurium to survive intracellularly and prevent clearance. Previous reports have suggested that the presence of IL-10 promotes the polarization of macrophages to the M2 phenotype (22), which suffer a metabolic change that increases the availability of intracellular glucose that, in turn, favor the intracellular replication of *S*. Typhimurium (62). In agreement with this idea, a previous study showed that, in macrophages, the production of the protein SpiC, which is encoded in SPI-2, induces the production of IL-10 in macrophages (29). Our results shown in Figure S4, Supplementary Material also support the notion that active replication of *S*. Typhimurium within innate immune cells increase the production of IL-10, because a higher amount of this cytokine is produced in DCs and BM macrophages infected with live bacteria, as compared to cells infected with heat-killed bacteria. The active production of IL-10 by infected phagocytic cells probably aids the intracellular survival of the bacteria and its dissemination from the intestine in early stages of the infection, favoring the colonization of other tissues, such as spleen and liver. Our results support this notion, since IL-10^−/−^ mice have less bacteria in ileum, spleen, and liver at 5 days of oral infection. Moreover, some of the IL-10^−/−^ mice evaluated in this study showed no bacteria in these tissues (Figure 1C).

In our experimental settings, the sole transfer of IL-10 producing DCs and BM macrophages did not restore the ability of *S*. Typhimurium to cause a systemic and lethal infection in IL-10^−/−^ mice. These results result suggests that the production of IL-10 that is required by *S*. Typhimurium to generate the systemic infection must be derived from adaptive immune cells. This statement is supported by our results showing that the transfer of IL-10-producing T cells recovered the ability of *S*. Typhimurium to cause a systemic infection in IL-10^−/−^ mice. Importantly, the sole presence of IL-10-producing T cells in infected IL-10^−/−^ mice restores the levels of IL-10 in serum observed in infected WT mice. These observations suggest that the generation of a tolerogenic, antigen-specific immune response driven by T cells is required by *S*. Typhimurium to cause systemic infection in C57BL/6 mice. Or results are in agreement with a previous report showing the relevance of Treg cells to sustain replication of *S*. Typhimurium in a murine model of bacterial persistence (35). In this study, which was performed in mice resistant to *S*. Typhimurium infection (F1 129SvJ X C57BL/6), it was described that depletion of FoxP3^+^ T cells resulted in reduced bacterial loads in the spleen after 3 weeks of *S*. Typhimurium infection. Similar results were observed when mice were treated with an anti-CLTA-4 antibody.

It is important to mention that, in our study, we observed a peak of IL-10 production at 5 days of infection, which agrees with the idea that the main producer of IL-10 are adaptive immune cells, or even by other innate immune cells that respond to signals provided by these adaptive immune cells. Using the IL-10/GFP VertX mice, we observed a significant increase of IL-10-producing DCs, monocytes, macrophages, and T cells in the ileum of infected mice when the peak of IL-10 production was detected. We also observed that resident DCs, monocytes, macrophages, and T cells in spleen probably increase their production of IL-10 upon *S*. Typhimurium infection. Moreover, we detected the presence of CD4^+^CD25^+^FoxP3^+^ T cells in ileum and spleen, which support the notion that Treg cells are being generated in *S*. Typhimurium-infected mice. It is important to mention that the detection of IL-10^+^ T cells in spleen observed in Figure 2, using IL-10/GFP VertX mice did not match the results found in Figure 3, was intracellular staining of IL-10 and FoxP3 was performed for T cells from WT-infected mice. It is important to consider that both models represent different ways to measure IL-10 production, and Figure 3 shows the results in a specific subset of T cells. Since the amount of Tregs in the tissues was quite low, it is expected that differences in sensitivity among both models account for the differences observed for IL-10-producing T cells in spleen. Furthermore, in the liver of the infected mice, we observed an increase of CD4^+^CD25^+^FoxP3^−^ T and increased expression of *ctla-4* and *pd-1*, but we did not observe an increase in the expression of *foxP3* in any tissue. Again, this assay should be read carefully, because total mRNA from each tissue was evaluated and the amount of CD4^+^CD25^+^FoxP3^+^ T cells increased in the ileum and spleen of infected mice is very low. Nevertheless, our result suggests that at the beginning of the systemic phase, Tregs cells are probably located in the intestine and liver, which agrees with previous reports showing that these tissues would be the preferred sites for Treg cells homing after infection with this and other pathogens (39, 63–69).

Another important finding in our study is the role of IL-10-producing B cell to restore the susceptibility to *S*. Typhimurium in IL-10^−/−^ mice. As shown in Figure 4, the transfer of IL-10-producing B cells increase the clinical score and enhance weight loss of infected IL-10^−/−^ mice, as compared to mice that receive IL-10-producing DCs and BM macrophages. The levels of mortality of these mice showed a slight increase, the bacterial loads were increased in the spleen, and these mice also showed increased levels of inflammatory cytokines in serum. However, these parameters were not equivalent in magnitude to those found in IL-10^−/−^ mice that received IL-10-producing T cells. This observation suggests that the production of IL-10 by B cells after *S*. Typhimurium infection reaches levels in the host that can support replication and dissemination of the bacterium. Indeed, it has been described that B cells can produce IL-10 after recognition of *S*. Typhimurium LPS through TLR-4, which is relevant to the early stages of the infection (21). However, in our experimental settings, we observed that the production of IL-10 by these cells cannot fully reverse the resistant of IL-10^−/−^ mice to the infection. Moreover, transfer of B cells did not increase the susceptibility of Rag.1^−/−^ mice to *S*. Typhimurium, while the transfer of T cells increased the susceptibility of these mice to the infection (Figures 4G,I). These results suggest that B cells may provide additional signals to T cells during *S*. Typhimurium infection that promotes the generation of Treg cells. In agreement with this idea, previous *in vitro* studies have shown that *S*. Typhimurium can infect and persist in B cells, plasma cells, and bone marrow precursors of B cells (70) and that infected cells express PD-L1 and PD-L2, which can promote the differentiation of T cells to Treg cells (71, 72). Therefore, the interplay between infected B cells and T cells can be key to the development of a tolerogenic immune response triggered by *S*. Typhimurium infection.

## Ethics Statement

All the experiments using mice were conducted in agreement with the ethical standards and according to the local animal protection law. All experimental protocols were reviewed and approved by the Scientific Ethical Committee for Animal and Environment Care of the Pontificia Universidad Católica de Chile (Protocol number CBB-208/2013).

## Author Contributions

GS, HP, CP-R, BS, FS, DP, NM-D, and RG performed the experiments. GS, HP, PG, AK, and SB analyzed the data. GS, HP, AK, and SB wrote the paper. MA-L and CR provided new materials and methodologies.

## Conflict of Interest Statement

The authors declare that the research was conducted in the absence of any commercial or financial relationships that could be construed as a potential conflict of interest.

## Abbreviations

*S*. Typhimurium, *Salmonella enterica* serovar Typhimurium; IL-10, interleukin-10; DCs, dendritic cells; BMM, bone marrow-derived macrophages; EGFP, enhanced green fluorescent protein; MOI, multiplicity of infection; Treg, regulatory T cells.

## References

[1] Ao TT Feasey NA Gordon MA Keddy KH Angulo FJ Crump JA Global burden of invasive nontyphoidal *Salmonella* disease, 2010(1). Emerg Infect Dis (2015) 21(6):941–9. 10.3201/eid2106.140999 PMC4451910 25860298

[2] Majowicz SE Musto J Scallan E Angulo FJ Kirk M O’Brien SJ The global burden of nontyphoidal *Salmonella* gastroenteritis. Clin Infect Dis (2010) 50(6):882–9. 10.1086/650733 20158401

[3] Scallan E Hoekstra RM Angulo FJ Tauxe RV Widdowson MA Roy SL Foodborne illness acquired in the United States – major pathogens. Emerg Infect Dis (2011) 17(1):7–15. 10.3201/eid1701.P21101 21192848 PMC3375761

[4] Gal-Mor O Boyle EC Grassl GA. Same species, different diseases: how and why typhoidal and non-typhoidal *Salmonella enterica* serovars differ. Front Microbiol (2014) 5:391. 10.3389/fmicb.2014.00391 25136336 PMC4120697

[5] Contini S. Typhoid intestinal perforation in developing countries: still unavoidable deaths? World J Gastroenterol (2017) 23(11):1925–31. 10.3748/wjg.v23.i11.1925 28373758 PMC5360633

[6] Gonzalez-Escobedo G Gunn JS. Gallbladder epithelium as a niche for chronic *Salmonella* carriage. Infect Immun (2013) 81(8):2920–30. 10.1128/IAI.00258-13 23732169 PMC3719562

[7] Gonzalez-Escobedo G Marshall JM Gunn JS. Chronic and acute infection of the gall bladder by *Salmonella typhi*: understanding the carrier state. Nat Rev Microbiol (2011) 9(1):9–14. 10.1038/nrmicro2490 21113180 PMC3255095

[8] Crawford RW Rosales-Reyes R Ramirez-Aguilar Mde L Chapa-Azuela O Alpuche-Aranda C Gunn JS. Gallstones play a significant role in *Salmonella* spp. gallbladder colonization and carriage. Proc Natl Acad Sci U S A (2010) 107(9):4353–8. 10.1073/pnas.1000862107 20176950 PMC2840110

[9] McArthur MA Fresnay S Magder LS Darton TC Jones C Waddington CS Activation of *Salmonella typhi*-specific regulatory T cells in typhoid disease in a wild-type S. Typhi challenge model. PLoS Pathog (2015) 11(5):e1004914. 10.1371/journal.ppat.1004914 26001081 PMC4441490

[10] Couper KN Blount DG Riley EM IL-10: the master regulator of immunity to infection. J Immunol (2008) 180(9):5771–7. 10.4049/jimmunol.180.9.5771 18424693

[11] Iyer SS Cheng G. Role of interleukin 10 transcriptional regulation in inflammation and autoimmune disease. Crit Rev Immunol (2012) 32(1):23–63. 10.1615/CritRevImmunol.v32.i1.30 22428854 PMC3410706

[12] Hedrich CM Bream JH. Cell type-specific regulation of IL-10 expression in inflammation and disease. Immunol Res (2010) 47(1–3):185–206. 10.1007/s12026-009-8150-5 20087682 PMC2892196

[13] Ma X Yan W Zheng H Du Q Zhang L Ban Y Regulation of IL-10 and IL-12 production and function in macrophages and dendritic cells. F1000Res (2015) 4(F1000 Faculty Rev):1465 10.12688/f1000research.7010.1

[14] Chaudhry A Samstein RM Treuting P Liang Y Pils MC Heinrich JM Interleukin-10 signaling in regulatory T cells is required for suppression of Th17 cell-mediated inflammation. Immunity (2011) 34(4):566–78. 10.1016/j.immuni.2011.03.018 21511185 PMC3088485

[15] Hsu P Santner-Nanan B Hu M Skarratt K Lee CH Stormon M IL-10 potentiates differentiation of human induced regulatory T cells via STAT3 and Foxo1. J Immunol (2015) 195(8):3665–74. 10.4049/jimmunol.1402898 26363058

[16] Bueno SM Gonzalez PA Carreno LJ Tobar JA Mora GC Pereda CJ The capacity of *Salmonella* to survive inside dendritic cells and prevent antigen presentation to T cells is host specific. Immunology (2008) 124(4):522–33. 10.1111/j.1365-2567.2008.02805.x 18266715 PMC2492944

[17] Bueno SM Tobar JA Iruretagoyena MI Kalergis AM. Molecular interactions between dendritic cells and *Salmonella*: escape from adaptive immunity and implications on pathogenesis. Crit Rev Immunol (2005) 25(5):389–403. 10.1615/CritRevImmunol.v25.i5.40 16167888

[18] Bueno SM Wozniak A Leiva ED Riquelme SA Carreno LJ Hardt WD *Salmonella* pathogenicity island 1 differentially modulates bacterial entry to dendritic and non-phagocytic cells. Immunology (2010) 130(2):273–87. 10.1111/j.1365-2567.2009.03233.x 20201987 PMC2878471

[19] Tobar JA Carreno LJ Bueno SM Gonzalez PA Mora JE Quezada SA Virulent *Salmonella enterica* serovar Typhimurium evades adaptive immunity by preventing dendritic cells from activating T cells. Infect Immun (2006) 74(11):6438–48. 10.1128/IAI.00063-06 17057096 PMC1695529

[20] Tobar JA Gonzalez PA Kalergis AM *Salmonella* escape from antigen presentation can be overcome by targeting bacteria to Fc receptors on dendritic cells. J Immunol (2004) 173(6):4058–65. 10.4049/jimmunol.173.6.4058 15356155

[21] Neves P Lampropoulou V Calderon-Gomez E Roch T Stervbo U Shen P Signaling via the MyD88 adaptor protein in B cells suppresses protective immunity during *Salmonella typhimurium* infection. Immunity (2010) 33(5):777–90. 10.1016/j.immuni.2010.10.016 21093317

[22] Lokken KL Mooney JP Butler BP Xavier MN Chau JY Schaltenberg N Malaria parasite infection compromises control of concurrent systemic non-typhoidal *Salmonella* infection via IL-10-mediated alteration of myeloid cell function. PLoS Pathog (2014) 10(5):e1004049. 10.1371/journal.ppat.1004049 24787713 PMC4006898

[23] Mooney JP Butler BP Lokken KL Xavier MN Chau JY Schaltenberg N The mucosal inflammatory response to non-typhoidal *Salmonella* in the intestine is blunted by IL-10 during concurrent malaria parasite infection. Mucosal Immunol (2014) 7(6):1302–11. 10.1038/mi.2014.18 24670425 PMC4177018

[24] Arai T Hiromatsu K Nishimura H Kimura Y Kobayashi N Ishida H Effects of in vivo administration of anti-IL-10 monoclonal antibody on the host defence mechanism against murine *Salmonella* infection. Immunology (1995) 85(3):381–8. 7558125 PMC1383910

[25] Belkaid Y Hoffmann KF Mendez S Kamhawi S Udey MC Wynn TA The role of interleukin (IL)-10 in the persistence of *Leishmania major* in the skin after healing and the therapeutic potential of anti-IL-10 receptor antibody for sterile cure. J Exp Med (2001) 194(10):1497–506. 10.1084/jem.194.10.1497 11714756 PMC2193677

[26] Eberhardt MK Deshpande A Fike J Short R Schmidt KA Blozis SA Exploitation of interleukin-10 (IL-10) signaling pathways: alternate roles of viral and cellular IL-10 in rhesus cytomegalovirus infection. J Virol (2016) 90(21):9920–30. 10.1128/JVI.00635-16 27558431 PMC5068540

[27] Redford PS Murray PJ O’Garra A. The role of IL-10 in immune regulation during *M. tuberculosis* infection. Mucosal Immunol (2011) 4(3):261–70. 10.1038/mi.2011.7 21451501

[28] Vansaun MN Mendonsa AM Lee Gorden D Hepatocellular proliferation correlates with inflammatory cell and cytokine changes in a murine model of nonalchoholic fatty liver disease. PLoS One (2013) 8(9):e73054 10.1371/journal.pone.0073054 24039859 PMC3767686

[29] Uchiya K Groisman EA Nikai T. Involvement of *Salmonella* pathogenicity Island 2 in the up-regulation of interleukin-10 expression in macrophages: role of protein kinase A signal pathway. Infect Immun (2004) 72(4):1964–73. 10.1128/IAI.72.4.1964-1973.2004 15039316 PMC375175

[30] Pie S Matsiota-Bernard P Truffa-Bachi P Nauciel C. Gamma interferon and interleukin-10 gene expression in innately susceptible and resistant mice during the early phase of *Salmonella typhimurium* infection. Infect Immun (1996) 64(3):849–54. 8641791 10.1128/iai.64.3.849-854.1996 PMC173847

[31] Foster GL Barr TA Grant AJ McKinley TJ Bryant CE MacDonald A Virulent *Salmonella enterica* infections can be exacerbated by concomitant infection of the host with a live attenuated S. enterica vaccine via Toll-like receptor 4-dependent interleukin-10 production with the involvement of both TRIF and MyD88. Immunology (2008) 124(4):469–79. 10.1111/j.1365-2567.2007.02798.x 18217948 PMC2492939

[32] Lee KS Jeong ES Heo SH Seo JH Jeong DG Choi YK. IL-10 suppresses bactericidal response of macrophages against *Salmonella typhimurium*. J Microbiol (2011) 49(6):1050–3. 10.1007/s12275-011-1043-z 22203573

[33] Nguyen T Robinson N Allison SE Coombes BK Sad S Krishnan L.IL-10 produced by trophoblast cells inhibits phagosome maturation leading to profound intracellular proliferation of *Salmonella enterica* Typhimurium. Placenta (2013) 34(9):765–74. 10.1016/j.placenta.2013.06.003 23834952 PMC3797447

[34] Shen P Roch T Lampropoulou V O’Connor RA Stervbo U Hilgenberg E IL-35-producing B cells are critical regulators of immunity during autoimmune and infectious diseases. Nature (2014) 507(7492):366–70. 10.1038/nature12979 24572363 PMC4260166

[35] Johanns TM Ertelt JM Rowe JH Way SS. Regulatory T cell suppressive potency dictates the balance between bacterial proliferation and clearance during persistent *Salmonella* infection. PLoS Pathog (2010) 6(8):e1001043. 10.1371/journal.ppat.1001043 20714351 PMC2920851

[36] Powell DA Roberts LM Ledvina HE Sempowski GD Curtiss R III Frelinger JA Distinct innate responses are induced by attenuated *Salmonella* enterica serovar Typhimurium mutants. Cell Immunol (2016) 299:42–9. 10.1016/j.cellimm.2015.10.002 26546408 PMC4704447

[37] Boonstra A Rajsbaum R Holman M Marques R Asselin-Paturel C Pereira JP Macrophages and myeloid dendritic cells, but not plasmacytoid dendritic cells, produce IL-10 in response to MyD88- and TRIF-dependent TLR signals, and TLR-independent signals. J Immunol (2006) 177(11):7551–8. 10.4049/jimmunol.177.11.7551 17114424

[38] Samarasinghe R Tailor P Tamura T Kaisho T Akira S Ozato K. Induction of an anti-inflammatory cytokine, IL-10, in dendritic cells after toll-like receptor signaling. J Interferon Cytokine Res (2006) 26(12):893–900. 10.1089/jir.2006.26.893 17238832

[39] Saraiva M O’Garra A. The regulation of IL-10 production by immune cells. Nat Rev Immunol (2010) 10(3):170–81. 10.1038/nri2711 20154735

[40] Sayi A Kohler E Toller IM Flavell RA Muller W Roers A TLR-2-activated B cells suppress *Helicobacter*-induced preneoplastic gastric immunopathology by inducing T regulatory-1 cells. J Immunol (2011) 186(2):878–90. 10.4049/jimmunol.1002269 21149607

[41] Medzhitov R TLR-mediated innate immune recognition. Semin Immunol (2007) 19(1):1–2. 10.1016/j.smim.2007.02.001 22228983 PMC3252746

[42] Hou B Benson A Kuzmich L DeFranco AL Yarovinsky F. Critical coordination of innate immune defense against *Toxoplasma gondii* by dendritic cells responding via their Toll-like receptors. Proc Natl Acad Sci U S A (2011) 108(1):278–83. 10.1073/pnas.1011549108 21173242 PMC3017180

[43] Casson CN Doerner JL Copenhaver AM Ramirez J Holmgren AM Boyer MA Neutrophils and Ly6Chi monocytes collaborate in generating an optimal cytokine response that protects against pulmonary *Legionella pneumophila* infection. PLoS Pathog (2017) 13(4):e1006309. 10.1371/journal.ppat.1006309 28384349 PMC5404877

[44] Mizuno Y Takada H Nomura A Jin CH Hattori H Ihara K Th1 and Th1-inducing cytokines in *Salmonella* infection. Clin Exp Immunol (2003) 131(1):111–7. 10.1046/j.1365-2249.2003.02060.x 12519393 PMC1808588

[45] Eckmann L Kagnoff MF. Cytokines in host defense against *Salmonella*. Microbes Infect (2001) 3(14–15):1191–200. 10.1016/S1286-4579(01)01479-4 11755407

[46] Ktsoyan Z Ghazaryan K Manukyan G Martirosyan A Mnatsakanyan A Arakelova K Inflammatory responses to *Salmonella* infections are serotype-specific. Int J Bacteriol (2013) 2013:168179. 10.1155/2013/168179 26904722 PMC4745457

[47] Chen KW Gross CJ Sotomayor FV Stacey KJ Tschopp J Sweet MJ The neutrophil NLRC4 inflammasome selectively promotes IL-1beta maturation without pyroptosis during acute *Salmonella* challenge. Cell Rep (2014) 8(2):570–82. 10.1016/j.celrep.2014.06.028 25043180

[48] Ma Y Chen H Wang Q Luo F Yan J Zhang XL. IL-24 protects against *Salmonella typhimurium* infection by stimulating early neutrophil Th1 cytokine production, which in turn activates CD8+ T cells. Eur J Immunol (2009) 39(12):3357–68. 10.1002/eji.200939678 19830736

[49] Pietila TE Veckman V Kyllonen P Lahteenmaki K Korhonen TK Julkunen I. Activation, cytokine production, and intracellular survival of bacteria in *Salmonella*-infected human monocyte-derived macrophages and dendritic cells. J Leukoc Biol (2005) 78(4):909–20. 10.1189/jlb.1204721 16033811

[50] Jensen K Gallagher IJ Kaliszewska A Zhang C Abejide O Gallagher MP Live and inactivated *Salmonella enterica* serovar Typhimurium stimulate similar but distinct transcriptome profiles in bovine macrophages and dendritic cells. Vet Res (2016) 47:46. 10.1186/s13567-016-0328-y 27000047 PMC4802613

[51] Spees AM Kingsbury DD Wangdi T Xavier MN Tsolis RM Baumler AJ. Neutrophils are a source of gamma interferon during acute *Salmonella enterica* serovar Typhimurium colitis. Infect Immun (2014) 82(4):1692–7. 10.1128/IAI.01508-13 24421037 PMC3993401

[52] Kupz A Scott TA Belz GT Andrews DM Greyer M Lew AM Contribution of Thy1+ NK cells to protective IFN-gamma production during *Salmonella typhimurium* infections. Proc Natl Acad Sci U S A (2013) 110(6):2252–7. 10.1073/pnas.1222047110 23345426 PMC3568339

[53] Jouanguy E Doffinger R Dupuis S Pallier A Altare F Casanova JL. IL-12 and IFN-gamma in host defense against mycobacteria and *Salmonella* in mice and men. Curr Opin Immunol (1999) 11(3):346–51. 10.1016/S0952-7915(99)80055-7 10375558

[54] Monack DM Bouley DM Falkow S. *Salmonella typhimurium* persists within macrophages in the mesenteric lymph nodes of chronically infected Nramp1+/+ mice and can be reactivated by IFNgamma neutralization. J Exp Med (2004) 199(2):231–41. 10.1084/jem.20031319 14734525 PMC2211772

[55] Bakowski MA Braun V Brumell JH. *Salmonella*-containing vacuoles: directing traffic and nesting to grow. Traffic (2008) 9(12):2022–31. 10.1111/j.1600-0854.2008.00827.x 18778407

[56] Steele-Mortimer O. The *Salmonella*-containing vacuole: moving with the times. Curr Opin Microbiol (2008) 11(1):38–45. 10.1016/j.mib.2008.01.002 18304858 PMC2577838

[57] Kolodziejek AM Miller SI. *Salmonella* modulation of the phagosome membrane, role of SseJ. Cell Microbiol (2015) 17(3):333–41. 10.1111/cmi.12420 25620407

[58] Huang J Brumell JH. Autophagy in immunity against intracellular bacteria. Curr Top Microbiol Immunol (2009) 335:189–215. 10.1007/978-3-642-00302-8_9 19802566

[59] Ertelt JM Johanns TM Mysz MA Nanton MR Rowe JH Aguilera MN Selective culling of high avidity antigen-specific CD4+ T cells after virulent *Salmonella* infection. Immunology (2011) 134(4):487–97. 10.1111/j.1365-2567.2011.03510.x 22044420 PMC3230801

[60] Rosales-Reyes R Alpuche-Aranda C Ramirez-Aguilar Mde L Castro-Eguiluz AD Ortiz-Navarrete V. Survival of *Salmonella enterica* serovar Typhimurium within late endosomal-lysosomal compartments of B lymphocytes is associated with the inability to use the vacuolar alternative major histocompatibility complex class I antigen-processing pathway. Infect Immun (2005) 73(7):3937–44. 10.1128/IAI.73.7.3937-3944.2005 15972480 PMC1168566

[61] van der Velden AW Copass MK Starnbach MN. *Salmonella* inhibit T cell proliferation by a direct, contact-dependent immunosuppressive effect. Proc Natl Acad Sci U S A (2005) 102(49):17769–74. 10.1073/pnas.0504382102 16306269 PMC1308886

[62] Eisele NA Ruby T Jacobson A Manzanillo PS Cox JS Lam L *Salmonella* require the fatty acid regulator PPARdelta for the establishment of a metabolic environment essential for long-term persistence. Cell Host Microbe (2013) 14(2):171–82. 10.1016/j.chom.2013.07.010 23954156 PMC3785333

[63] Atarashi K Tanoue T Shima T Imaoka A Kuwahara T Momose Y Induction of colonic regulatory T cells by indigenous *Clostridium* species. Science (2011) 331(6015):337–41. 10.1126/science.1198469 21205640 PMC3969237

[64] Honda K Littman DR The microbiome in infectious disease and inflammation. Annu Rev Immunol (2012) 30:759–95. 10.1146/annurev-immunol-020711-074937 22224764 PMC4426968

[65] Maynard CL Harrington LE Janowski KM Oliver JR Zindl CL Rudensky AY Regulatory T cells expressing interleukin 10 develop from Foxp3+ and Foxp3- precursor cells in the absence of interleukin 10. Nat Immunol (2007) 8(9):931–41. 10.1038/ni1504 17694059

[66] Hayashi A Sato T Kamada N Mikami Y Matsuoka K Hisamatsu T A single strain of *Clostridium butyricum* induces intestinal IL-10-producing macrophages to suppress acute experimental colitis in mice. Cell Host Microbe (2013) 13(6):711–22. 10.1016/j.chom.2013.05.013 23768495

[67] Herkel J. Regulatory T cells in hepatic immune tolerance and autoimmune liver diseases. Dig Dis (2015) 33(Suppl 2):70–4. 10.1159/000440750 26642346

[68] Riezu-Boj JI Larrea E Aldabe R Guembe L Casares N Galeano E Hepatitis C virus induces the expression of CCL17 and CCL22 chemokines that attract regulatory T cells to the site of infection. J Hepatol (2011) 54(3):422–31. 10.1016/j.jhep.2010.07.014 21129807

[69] Lapierre P Janelle V Langlois MP Tarrab E Charpentier T Lamarre A Expression of viral antigen by the liver leads to chronic infection through the generation of regulatory T cells. Cell Mol Gastroenterol Hepatol (2015) 1(3):325–41.e1. 10.1016/j.jcmgh.2015.02.002 28210682 PMC5301191

[70] Castro-Eguiluz D Pelayo R Rosales-Garcia V Rosales-Reyes R Alpuche-Aranda C Ortiz-Navarrete V. B cell precursors are targets for *Salmonella* infection. Microb Pathog (2009) 47(1):52–6. 10.1016/j.micpath.2009.04.005 19383536

[71] Lopez-Medina M Carrillo-Martin I Leyva-Rangel J Alpuche-Aranda C Ortiz-Navarrete V. *Salmonella* impairs CD8 T cell response through PD-1: PD-L axis. Immunobiology (2015) 220(12):1369–80. 10.1016/j.imbio.2015.07.005 26210046

[72] Lopez-Medina M Perez-Lopez A Alpuche-Aranda C Ortiz-Navarrete V. *Salmonella* induces PD-L1 expression in B cells. Immunol Lett (2015) 167(2):131–40. 10.1016/j.imlet.2015.08.004 26292028

