# Supplementary material for: Interleukin-10 Production by T and B Cells Is a Key Factor to Promote Systemic Salmonella enterica Serovar Typhimurium Infection in Mice
Source: Front Immunol. 2017 Aug 2;8:889. doi: 10.3389/fimmu.2017.00889 (PMC5539121; doi:10.3389/fimmu.2017.00889)
Supplement: Supplementary file 1 [file data_sheet_1.pdf]

LEGENDS FOR SUPPLEMENTARY FIGURES

Supplementary Figure S1. Gating strategy to identify myeloid IL-10 producing cells. To identify each myeloid cell population, total events were selected by the singlets and debris. Then, viable cells were selected using the fixable viability stain 510 dye (BD) and $CD45^+$ live cells were gated. From this population two different gates were performed. First, $CD11b^+$ cells were selected to identify neutrophils. From Ly6G negative cells, $CD11b^+$SSC-AlowSiglecF- were selected to identify monocytes and then dendritic cells and macrophages. Each cell population was quantified using count bright beads. From $CD45^+$ cells we also gated the $CD45^+GFP^+$ cells (*). From this gate, we identified and quantified the number of each GFP^+ cell type using the same strategy explained below.

Supplementary Figure S2. Gating strategy to identify lymphoid IL-10 producing cells. To identify each lymphoid cell population, total events were selected by the singlets and debris. Then, viable cells were selected using the fixable viability stain 510 dye (BD) and $CD45^+$ live cells were gated. From this population two different gates were performed. First, CD3 and CD19 were used to differentiate T cells and B cells. On $CD3^+$ cells, $CD4^+$ T cells and $CD8^+$ T cells were identified. From $CD19^+$ cells, CD1d was used to identified $CD1d^+$ and $CD1d^-$ B cells. Likewise, in myeloid cells, from $CD45^+$ cells the total GFP^+ cells were gated and using the same gate strategy explained below (*), B and T GFP^+ cells were identified and quantified using count bright beads.

Supplementary Figure S3. Gating strategy to identify Treg cells. (A) To identify Treg cell population, total events were selected by the singlets and debris. Gates were set on total live cells, then $CD45^+$ cells. $CD45^+$ cells were identified for TCR^+ to differentiate T cells. $CD4^+CD25^+$ T cells were separated into $Foxp3^+$ Tregs and $Foxp3^-$ T conventional cells. All these cells were identified and quantified using count bright beads. **(B)** Histograms show representative expression of IL-10 gated on live CD4 $^+CD25^+$, $CD4^+CD25^+Foxp3^+$ and $CD4^+CD25^+Foxp3^-$ lymphocytes in uninfected mice (gray line) or *S*. Typhimurium infected mice (black line).

Supplementary Figure S4. IL-10 is produced *in vitro* by immune cells after *S*. Typhimurium infection. Dendritic cells (DCs), bone marrow derived (BM) macrophages, B cells and T cells were purified from WT C57BL/6 as described in materials and methods and infected with live or heat killed (HK) *S*. Typhimurium (ST), MOI 25, for 2 h. Then, gentamicin treatment was performed to eliminate extracellular bacteria and the antibiotic treated cells were incubated for additional 22 h. The secretion of IL-10 to the media was evaluated by ELISA Kit II (BD Biosciences). As a negative control, uninfected-unstimulated DCs, BM macrophages, B cells and T cells were included in the experiment (UT). As positive controls for IL-10 production by dendritic cells, BM macrophages and B cells, treatment with LPS (1 µg/ml, *Salmonella* Typhimurium; Sigma, St Louis, MO) was included. As additional positive control for IL-10 production by B cells, treatment with IgM (10 µg/ml) and IgM+LPS was included for 24 h. As a positive control for IL-10 production by T cells, a treatment with antibodies α -CD3/α -CD28 (1 µg/ml) was included. Differences among the treatments was assessed by t-student test between negative controls and treatments. *$P<0.05$, **$P<0.01$; ***$P<0.001$.
