# Supplementary figures and images for: Interleukin-10 Production by T and B Cells Is a Key Factor to Promote Systemic Salmonella enterica Serovar Typhimurium Infection in Mice
Source: Front Immunol. 2017 Aug 2;8:889. doi: 10.3389/fimmu.2017.00889 (PMC5539121; doi:10.3389/fimmu.2017.00889)

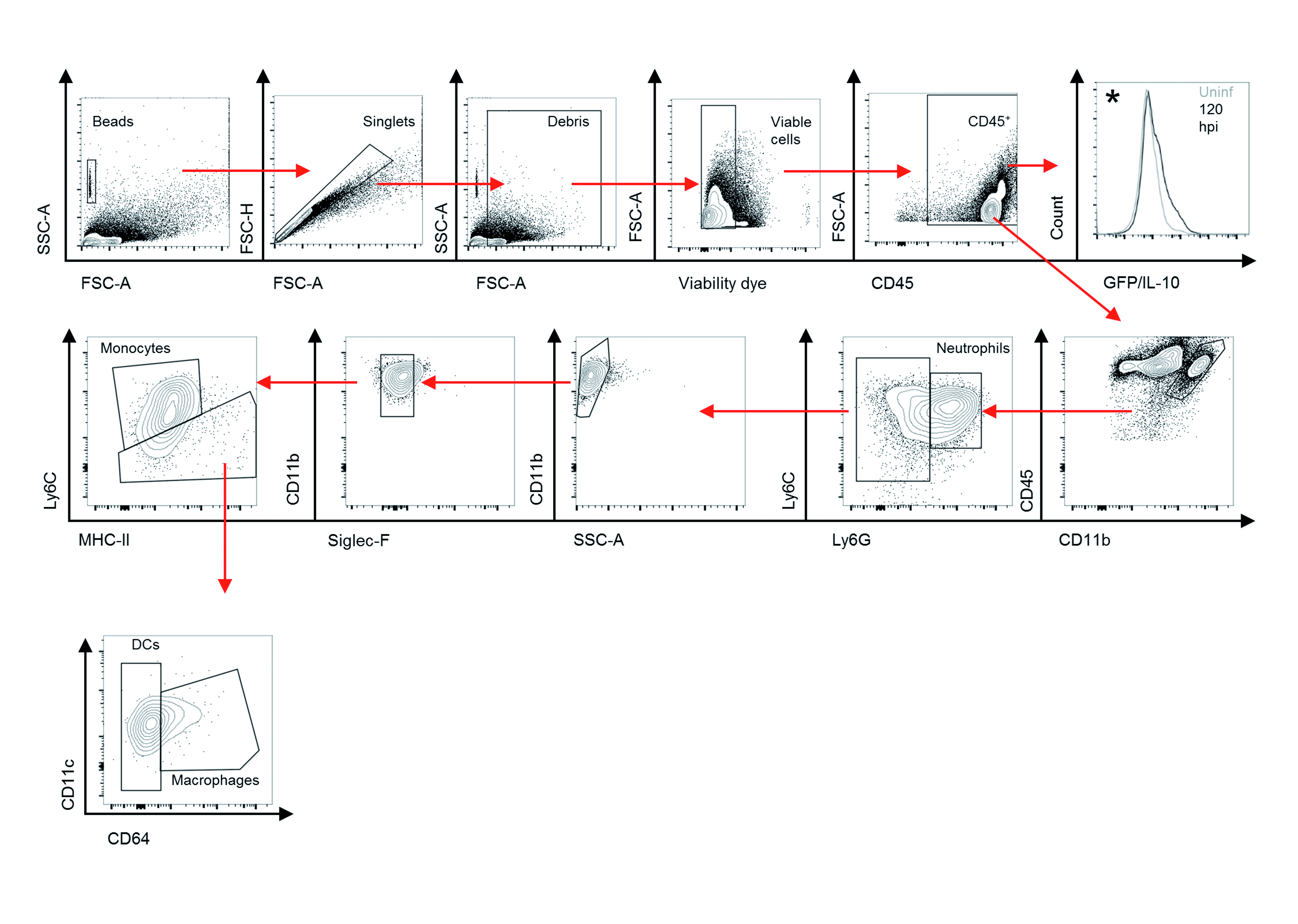

Supplement: Supplementary file 2 [file image_1.tif]

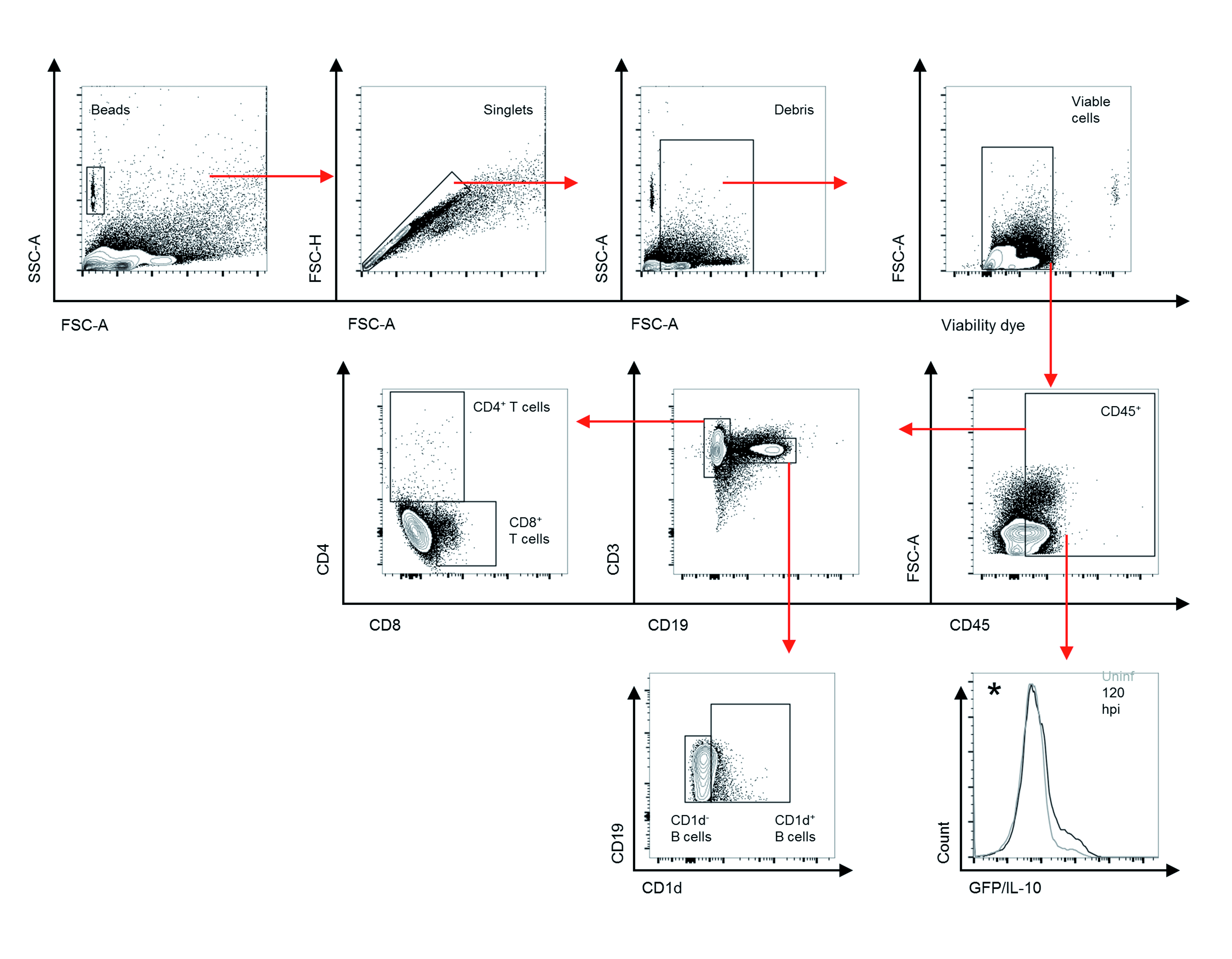

Supplement: Supplementary file 3 [file image_2.tif]

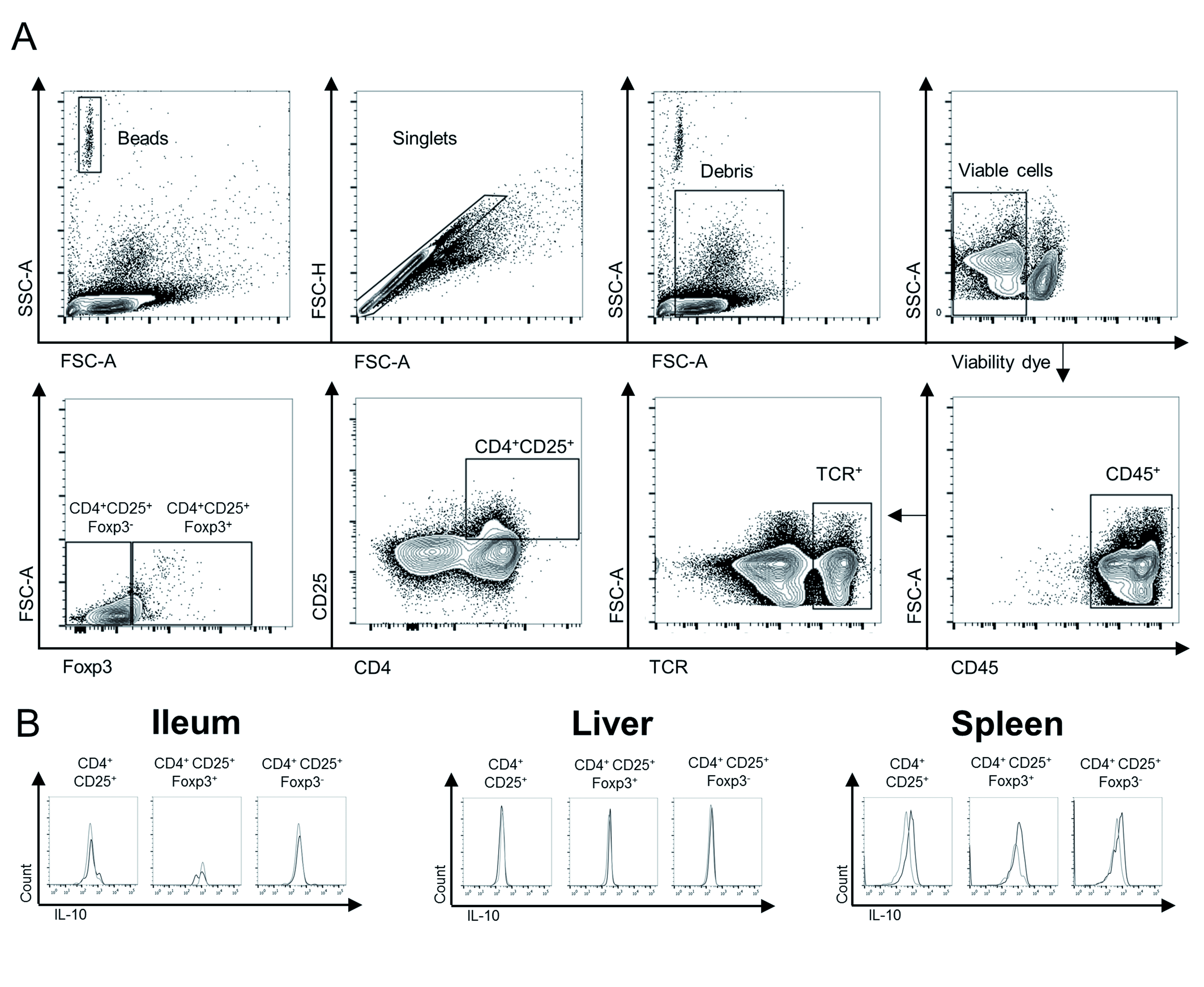

Supplement: Supplementary file 4 [file image_3.tif]

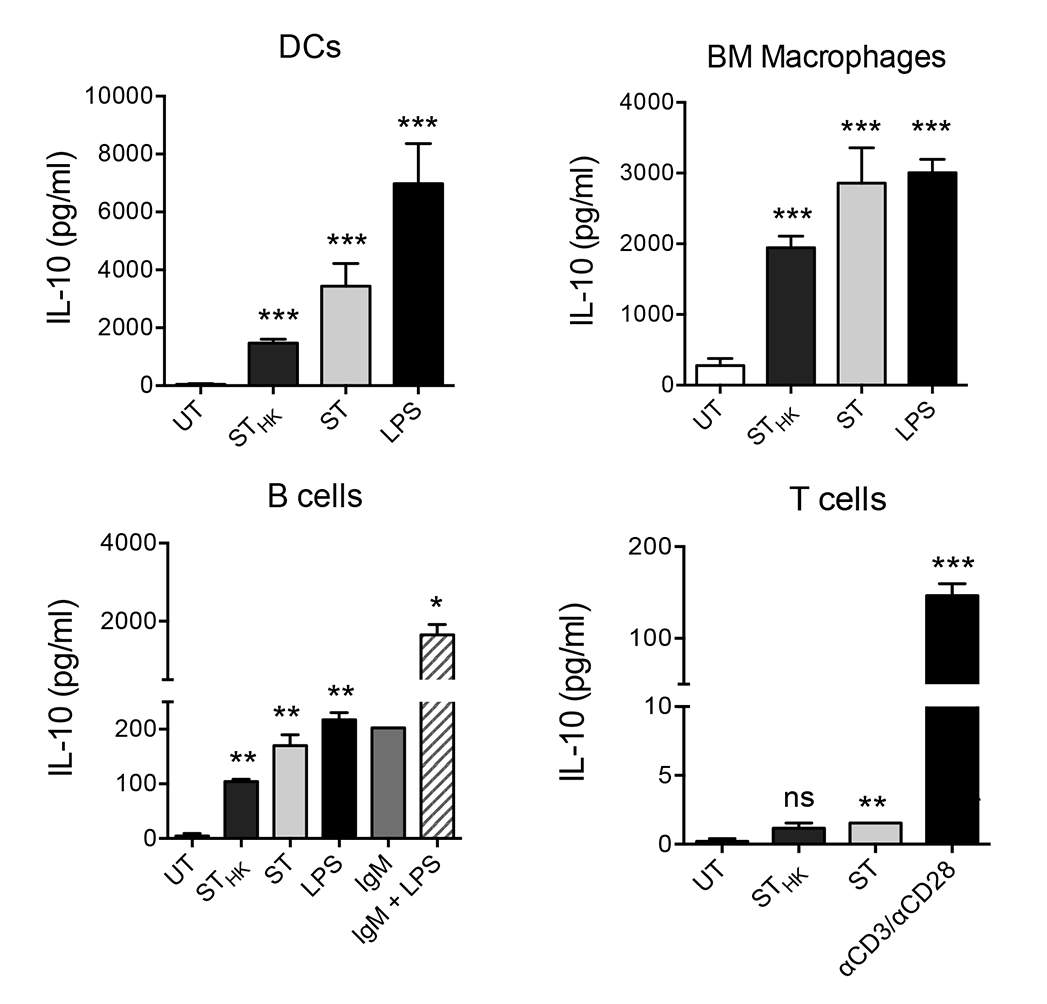

Supplement: Supplementary file 5 [file image_4.tif]
