# Supplementary material for: Interleukin-10 Production by T and B Cells Is a Key Factor to Promote Systemic Salmonella enterica Serovar Typhimurium Infection in Mice
Source: Front Immunol. 2017 Aug 2;8:889. doi: 10.3389/fimmu.2017.00889 (PMC5539121; doi:10.3389/fimmu.2017.00889)
Supplement: Supplementary file 6 [file table_1.docx]

**TABLE S1. CLINICAL AND PHYSIOLOGICAL SCORE TO EVALUATED DISEASES ACTIVITY INDEX (DAI) DURING A *SALMONELLA* INFECTION.**

The aim of this score is evaluated and recorded, on daily basis, health and wellness conditions during an infective process to avoid unnecessary suffering on the experimental mice.

| Parameter | Observations | Score |
| --- | --- | --- |
| **PHYSIOLOGICAL INDEX**  -Weight Loss  -Body temperature  (measure on abdominal surface) | Normal: No weight loss, Body temperature (T°) between 36,5-38,0°C.  Mild: Weight lost less than 10%, T°=34,5°C-36,4°C  Moderate: Weight loss between 10-20%, T°=34,5-36,4°C or 38,6-39.5°C  Severe: Weight loss over 20%. T°<34,5 or >39.5°C | 0  1  2  3 |
| **GENERAL CONDITION** | Normal: Mice are active, moved around the cage, grooming, shiny hair.  Mild: Reluctance to move, Restlessness,  piloerection, rough hair, Porphyrin discharge (red-brown pigment around eyes and nostrils)  Moderate: Weak attitude, hunched posture, dull or sluggish movements.  Severe: Extreme weakness, mouse that does not move on the cage, sunken eyes and severe dehydration | 0  1  2  3 |
| **BEHAVIOR** | Normal: Alert attitude, grooming, eating, drinking and interacting with cage mates.  Mild: Reduce grooming and movements into the cage, signs of tachypnea, remains into the bottom of cage.  Moderate: Depression, lethargic, uncoordinated movements on cage, poor grooming, abdominal breathing.  Severe: Extreme lethargy, prostration, panting, no grooming and fuzzy facial fur. | 0  1  2  3 |

**DAI:**

- 0 a 3: Normal
- 4 a 6: Careful monitoring.
- 7 a 9: Signs of severe pain, euthanasia would be considered
- 10 a 12: Final Point criteria, Euthanasia would be perform.
